# Identification of Rice Varieties and Transgenic Characteristics Based on Near-Infrared Diffuse Reflectance Spectroscopy and Chemometrics

**DOI:** 10.3390/molecules24244568

**Published:** 2019-12-13

**Authors:** Yong Hao, Pei Geng, Wenhui Wu, Qinhua Wen, Min Rao

**Affiliations:** 1 School of Mechatronics & Vehicle Engineering, East China Jiaotong University, Nanchang 330013, China; gengpei29@163.com (P.G.); wwh191686977wwh@163.com (W.W.); wenqinhua1@163.com (Q.W.); 2 Ganzhou Entry-Exit Inspection and Quarantine Bureau, Ganzhou 341000, China; raomin2233@163.com

**Keywords:** portable near-infrared reflectance spectroscopy (NIRDRS), rice varieties, transgenic rice, partial least squares discriminant analysis (PLS-DA), support vector machines (SVM)

## Abstract

Background: In recent years, genetically modified technology has developed rapidly, and the potential impact of genetically modified foods on human health and the ecological environment has received increasing attention. The currently used methods for testing genetically modified foods are cumbersome, time-consuming, and expensive. This paper proposed a more efficient and convenient detection method. Methods: Near-infrared diffuse reflectance spectroscopy (NIRDRS) combined with multivariate calibration methods, including principal component analysis (PCA), partial least squares discriminant analysis (PLS-DA), and support vector machines (SVM), were used for identification of different rice varieties and transgenic (Bt63)/non-transgenic rice. Spectral pretreatment methods, including Norris–Williams smooth (NWS), standard normal variate (SNV), multiplicative scatter correction (MSC), and Savitzky–Golay 1st derivative (SG 1st-Der), were used for spectral noise reduction and effective information enhancement. Accuracy was used to evaluate the qualitative discriminant models. Results: The results showed that the SG 1st-Der pretreatment method, combined with the SVM, provided the optimal model to distinguish different rice varieties. The accuracy of the optimal model was 98.33%. For the discrimination model of transgenic/non-transgenic rice, the SNV-SVM model, MSC-SVM model, and SG 1st-Der-PLS-DA model all achieved good analysis results with the accuracy of 100%. Conclusion: The results showed that portable NIR spectroscopy combined with chemometrics methods could be used to identify rice varieties and transgenic characteristics (Bt63) due to its fast, non-destructive, and accurate advantages.

## 1. Introduction

Genetically modified organisms (GMOs), also known as genetically engineered (GE) or transgenic organisms, are often used to modify human foods and animal feeds nowadays. A total of 28 countries cultivate transgenic plants, and the planted area has been expanded 100 times to 181.5 million hectares since 1996 [1]. The transgenic technology has become a potential tool for addressing the world food crisis while, at the same time, posing potential risks to human health, the environment, and biodiversity [2,3]. The public is skeptical about the safety of GMOs; therefore, transgenic technology is banned in many countries of the world. In recent years, in China, the Ministry of Agriculture intends to strengthen research on the safety assessment, regulation, and management of agricultural GMOs. In order to standardize the introduction process and production of GMOs, fast and reliable detection methods are urgently needed.

Rice is one of the most important crops in the world. The transgenic technology has been applied to increase rice production and improve rice quality. In 2009, the Chinese Agriculture Ministry awarded the GM rice Bt-Shanyou63 (Bt-SY63) a safety certificate. *Bacillus thuringiensis* (BT) proteins are the most widely used insecticidal proteins in transgenic crops for improving insect resistance [4]. Bt63 transgenic rice has strong insect resistance and can greatly reduce the use of pesticides. Despite this, the debate over its safety has continued ever since.

The various methodologies have been employed to detect the GMOs. These methods are mostly based on DNA or protein analysis, such as enzyme-linked immunosorbent assays (ELISAs), lateral flow strip, biosensor, western blot, real-time PCR, qualitative polymerase chain reaction (qPCR), microarray, electrophoresis, southern blot, liquid chromatography, and gas chromatography [5,6,7,8,9,10]. However, these technologies have many disadvantages, including high cost, difficulty to use, special needs, time-consuming analysis, and so on.

Near-infrared diffuse reflectance spectroscopy (NIRDRS), which is non-destructive, simple, fast, has lower cost, and no or simple sample pretreatment, has become a research hotspot for many scholars. It is difficult to detect the modified GMOs through DNA analysis because its contents are ultra-trace. However, it is feasible to use proteins or larger structural changes analysis to detect GMOs products [11,12]. In the previous studies, NIRS has been successfully used in the detection of transgenic corns, tomato, and soybean oils [13,14].

The purpose of the present study was to apply the portable NIRDRS to discriminate rice varieties and transgenic characteristics. Principal component analysis (PCA), partial least squares discriminant analysis (PLS-DA), and support vector machines (SVM) were employed to build the discrimination models. It was expected to propose a rapid identification method for rice varieties and transgenic characteristics.

## 2. Results

### 2.1. Diffuse Reflectance Spectra of Rice Grains

**Figure 1:**
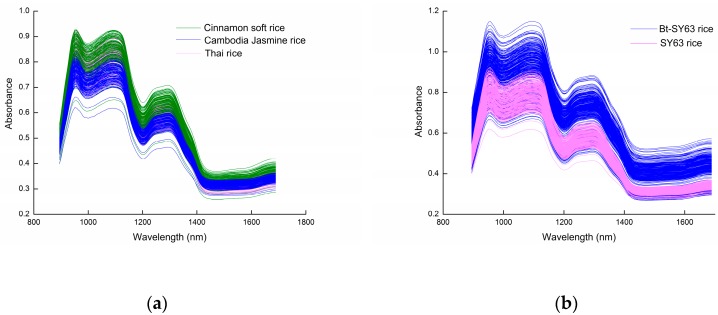
The original spectra of rice species and transgenic and non-transgenic. (**a**) The original spectra of rice species; (**b**) The original spectra of SY63 and Bt-SY63 rice.

The original spectra of the Cambodia Jasmine rice, Thai rice, Cinnamon soft rice, SY63, and Bt-SY63 rice are shown in Figure 1a,b. The original spectra were the spectra without any pretreatment. The spectra of rice samples were overlapped and similar. This was because the main component of rice is starch. Therefore, for rice samples of different varieties and genetically modified samples, the main reaction of the near-infrared spectrum was starch, and the spectra were very similar. It was impossible to distinguish species and transgenic characteristics from each other only from the spectra. Therefore, it was necessary to employ spectral pretreatment and qualitative discrimination methods to distinguish varieties and transgenic characteristics clearly.

### 2.2. PCA of Rice Spectra

**Figure 2:**
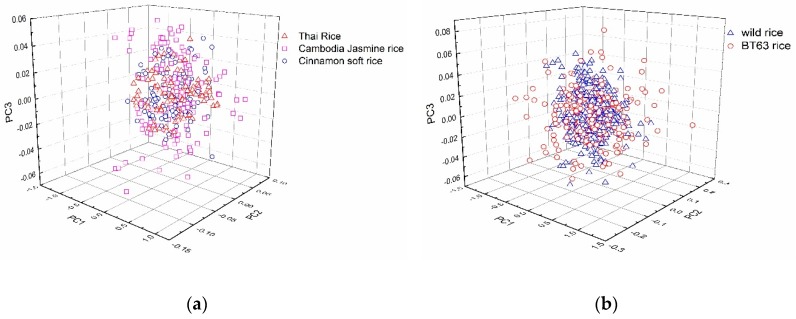
The results of PCA (principal component analysis). (**a**) The PCA of three types of non-transgenic rice grains; (**b**) The PCA of transgenic and non-transgenic rice grains.

The PCA was performed on the original spectra, and the first three principal component distributions of the sample spectra are shown in Figure 2a,b. The spectra of Cambodia Jasmine rice (Pink square), Thai rice (red triangle), and Cinnamon soft rice (blue circle) were overlapped (Figure 2a) and could not be separated adequately. It was also difficult to identify the transgenic (red circle) and non-transgenic (blue square) rice directly from the spatial distribution of the sample spectra (Figure 2b). Although PCA could extract useful spectral information and reduce spectral dimensions, it was still hard to effectively distinguish the attribution of samples. The supervised pattern recognition methods were still needed for the establishment of a qualitative discrimination model.

### 2.3. PLS-DA Model Establishment and Analysis

The PLS-DA models were built to identify rice varieties and transgenic/non-transgenic rice. Leave-one-out cross-validation (LOOCV), combined with the *f*-test method, was used to determine the principal factors of the PLS-DA model for avoiding overfitting or underfitting [15]. The PLS-DA models of original and preprocessing spectra were built for the identification of rice varieties, and the statistical results of the models are shown in Table 1.

The original spectral model had an accuracy of 95.833%. The discrimination effect of spectral pretreatment by SNV (standard normal variate), MSC (multiplicative scatter correction), and SG 1st-Der (Savitzky–Golay 1st derivative) was not good. The accuracy of SNV and MSC was even lower than that of without pretreatment. Although the accuracy of SG 1st-Der was the same as the original spectra, the number of LVs (latent variables) was increased from 8 to 11. The NWS (Norris–William smooth) method obtained the best analytical accuracy, and the accuracy was improved to 97.5% with LVs of 14. In Table 2, the results of the PLS-DA model for discrimination of transgenic and non-transgenic rice are shown in detail. Obviously, due to the spectral complexity for containing transgenic elements, the number of LVs in each model were larger than those in Table 1. The accuracy of the original spectral model was 98.75%. The SNV, MSC, and SG 1st-Der preprocessing could provide a better model with an accuracy of 100%, but the SG 1st-Der provided the optimal model with the less LVs.

### 2.4. SVM Model Establishment and Analysis

The SVM model was applied in the discrimination of three kinds of rice and transgenic and non-transgenic rice, and the optimal solution of C and gamma was obtained by the PSO (particle swarm optimization) algorithm. The results of the optimal parameters C and gamma are shown in Table 3 and Table 4. The optimal parameters changed with different spectral pretreatment methods.

When the SVM model was applied to the discrimination of three kinds of rice, the accuracy of the model established using the original spectra was 92.70% (Table 3). SNV, MSC, and SG 1st-Der all contributed to the performance of the SVM model. SG 1st-Der-SVM model had the best accuracy, and the accuracy was increased to 99.58%.

The SVM model had a good performance in the discrimination of the transgenic and non-transgenic rice, both in the original spectrum and the spectrum processed by different pretreatment methods (Table 4). The accuracy of the original spectrum could reach 99.38%; both the SNV-SVM model and MSC-SVM model achieved optimal analysis results with the accuracy of 100%.

### 2.5. Selection of Optimal Discriminant Model

For the identification of rice varieties, the accuracy of the SG 1st-Der-SVM model was better than that of the NWS-PLS-DA model. Therefore, the SG 1st-Der-SVM model was used for the identification of rice varieties. For the identification of transgenic and non-transgenic rice, SG 1st-Der-PLS-DA, SNV-SVM, and MSC-SVM models could all achieve the accuracy of 100%. In order to simplify the construction process of the model and keep consistent with the construction of the method of the rice variety identification model, the SVM method was selected for the identification of transgenic characteristics. Compared with the MSC pretreatment method, the SNV method did not need to calculate the average spectrum as the ideal target spectrum; therefore, the SNV-SVM model was adopted for the identification of transgenic and non-transgenic rice.

**Figure 3:**
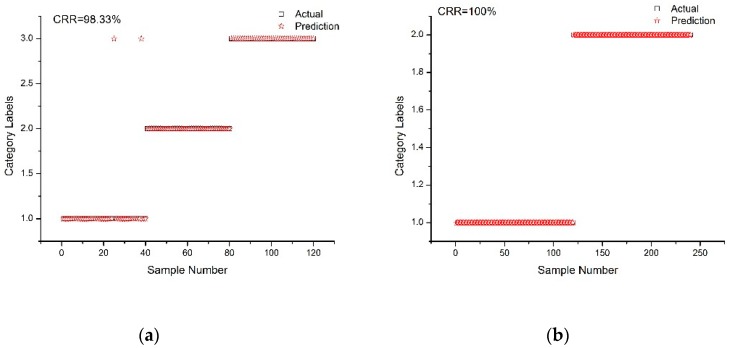
(**a**) Prediction effect of SG 1st-Der-SVM (Savitzky–Golay 1st derivative-support vector machines) model for the identification of rice varieties; (**b**) Prediction effect of SNV-SVM (standard normal variate-support vector machines) model for the identification of transgenic and non-transgenic rice.

The prediction results of the optimal SVM model on rice varieties and transgenic and non-transgenic rice are shown in Figure 3a,b. The accuracy of the optimal rice variety model was 98.33% (Figure 3a), and the accuracy of the optimal transgenic/non-transgenic rice model was 100% (Figure 3b).

## 3. Discussion

**Figure 4:**
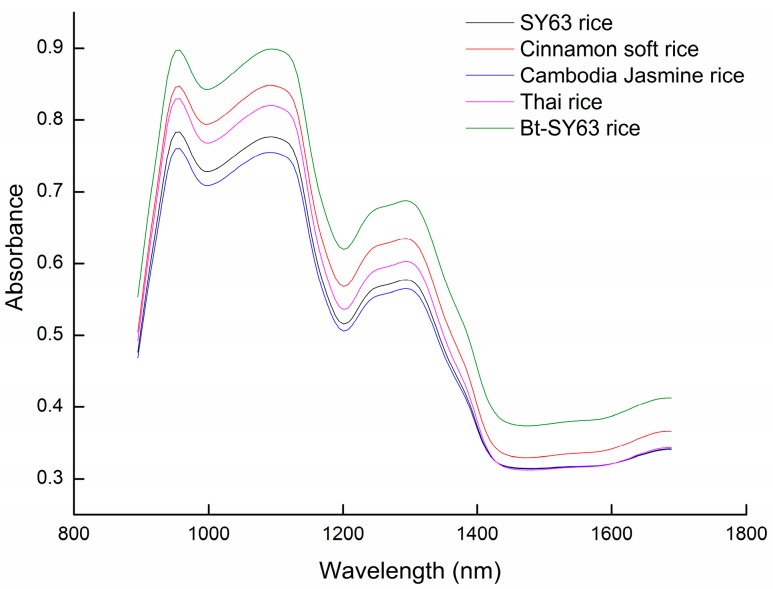
The average spectra of rice species and transgenic/non-transgenic rice.

By comparing the results of different modeling methods on rice varieties and transgenic characteristics, it could be seen that the accuracies of transgenic characteristics discrimination models were superior to those of rice species discrimination models. The average spectra of rice varieties and transgenic/non-transgenic rice are shown in Figure 4. It could be seen from Figure 4 that in the range of wavelengths of 950 to 1150 nm and 1200 to 1300 nm, there were significant offsets in the spectral absorbance of various types of rice, as well as transgenic and non-transgenic. Among them, 1200 to 1300 nm is a glucose absorption band [16,17].

The main component of rice is starch. Rice starch is a polysaccharide polymer compound composed of glucose, which contains amylopectin mainly composed of a branched structure and amylose mainly composed of a linear structure [18,19]. Due to the influence of geographical location, the rice’s starch content, molecular weight, spatial structure, and their relationship are different. The principal characteristic of BT is the synthesis, during sporulation, of a crystalline inclusion containing proteins known as Cry proteins. These proteins have insecticidal properties [20]. In the near-infrared region, the absorption of protein is quite different from that of starch [21,22,23,24]. The absorption peaks associated with the first overtone of C-H stretching of the starch are around 1131–1155 nm, and the protein and combination bands are around 1950–2250 nm [25]. Therefore, the better recognition effect of the transgenic model may be due to the introduction of BT protein.

## 4. Material and Methods

### 4.1. Rice Samples

**Figure 5:**
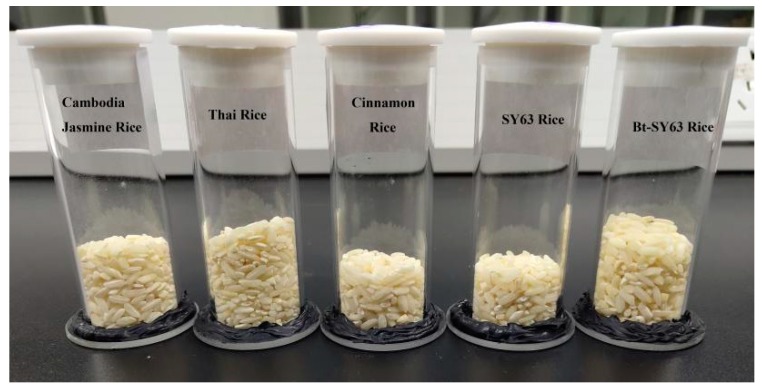
Samples and spectral acquisition accessories (container).

A total of 360 rice samples, including Cambodia Jasmine rice, Thai rice, and Cinnamon soft rice, were used to identify the species, and each type of rice contains 120 samples. A total of 720 rice samples, including genetically modified rice Bt Shanyou 63 (Bt-SY63) and its non-transgenic isogenic counterparts Shanyou63 (SY63), were used to discriminate transgenic (Bt63)/non-transgenic characteristics; both transgenic and non-transgenic rice samples were 360 in total (Table 5) (Details please see the Supplementary Materials). Bt genes were expressed in rice plants, and the expression levels were stable. All samples were provided by the Center of Science and Technology Development, Ministry of Agriculture of the People’s Republic of China. Some samples and spectral acquisition accessories (container) are shown in Figure 5.

### 4.2. Spectral Measurements

The NIRDRS of rice samples was scanned by using the MicroNIR spectrometer (JDSU, Milpitas, CA, USA), which owes its small size for using the novel thin-film linearly variable filter (LVF) as the dispersive element. All spectra were collected from 900 to 1700 nm in diffuse reflectance mode with a self-developed software system by MatlabR2014a (MathWorks, Natick, MA, USA). The gold body was used to collect the reference spectrum and calibrate the background. The spectral resolution was digitalized with ca. 6 nm, and each spectrum was composed of 128 data points. When collecting spectra, the temperature was balanced at 26 °C, and the humidity was 51%. In order to overcome the optical path difference of near-infrared light transmission caused by incompact sample packing, the samples were reloaded, and the container was shaken at each spectra acquisition. Three spectra were collected for each sample after each loading, and the average spectrum was used as the final analytical spectrum.

### 4.3. Spectral Data Pretreatment

For solid samples, due to the optical path difference and scattering effect, there would be rotation and translation errors in NIRDRS. Effective data pretreatment methods were used to reduce spectral noise and enhance effective information. In this study, several different spectral pretreatment methods, including Norris–Williams smooth (NWS), standard normal variate (SNV), multiplicative scatter correction (MSC), and Savitzky–Golay 1st derivative (SG 1st-Der), were carried out to improve spectral resolution and signal to noise ratio [26,27].

### 4.4. Modeling Methods

Near-infrared spectra (NIR) is a wide-band response. Therefore, the qualitative and quantitative analysis of substances should be carried out by means of chemometrics, e.g., PCA, PLS-DA, and SVM.

In this study, the original spectra were divided into a calibration set and test set according to the ratio of 2:1 by Kennard–Stone method [28]. In order to discriminate the three types of rice, each kind of rice had 80 spectra for model training and another 40 for testing. Thus, the calibration set sample had 240, and the prediction set sample had 120. In the discrimination of transgenic and non-transgenic rice, the 720 spectra were divided into two parts in the same way. Therefore, each category had 240 spectra in the training set and another 120 spectra in the test set.

PCA is a method for reducing high dimension data set by a decomposing linear combination of original variables into a few principal components [29,30]. In consequence, PCA allows visualization of natural clustering in the data. Each raw spectrum can be then represented as a linear combination of factors and its eigenvalue. The principal component factor number is the most important parameter of the method for further analysis. PCA of the original spectra or the pretreated data can provide very important information about object separation.

The PLS-DA model is developed based on the PLS algorithm, which is a sophisticated multivariate regression model and can establish mathematical relationships between descriptors and dependent variables [31,32]. PLS algorithm obtains latent variables (LVs) by linear combination of original variables and ranks the LVs. Among the LVs after sorting, the most advanced LVs contain the largest amount of useful information. The performance of the PLS model is mainly affected by the number of LVs [33]. Determining the number of LVs is the key to establish the PLS model. The PLS can be used for discrimination analysis by designing the values of different categories. In order to build the discriminant model of rice species, the labels of Cambodia Jasmine rice, Thai rice, and Cinnamon soft rice were designated as ‘1′, ’2′, ’3′, respectively. The labels of Bt63 rice and non-transgenic rice were designated as ‘1′ and ’2′ to discriminate genetically modified characteristics of rice.

SVM proposed by Vapnik in 1995 is a statistical-based machine learning, which was first used to identify postal code handwriting [34,35,36]. SVM can be applied to perform nonlinear classification on a large number of high-dimensional data with good generalization performance. These are the main features that the SVM method is superior to other linear chemometrics analysis methods, such as PLS.

In the establishment of the SVM model, the radial basis function (RBF) was selected as the kernel function in the study. Its basic expression is shown in Equation (1). The performance of the RBF kernel is determined by the penalty factor (C) and the parameters *gamma* [37]. C represents the tolerance of the model to the error. The model’s tolerance to error is too small, and the model is easy to overfit when C is too large. Conversely, the model is prone to underfitting when C is too small. Improper C will lead to poor generalization ability of the model. The expression of gamma is shown in Equation (2).

(1) $$k\left(x_{i},y_{i}\right)=exp\left(-\frac{\|x_{1}-x_{2}{\|}^{2}}{2\sigma^{2}}\right)$$

(2) $$gamma=\frac{1}{2\sigma^{2}}$$

The particle swarm optimization (PSO) was used to obtain the optimal solutions for C and gamma, which was proposed by Kennedy and Eberhart in 1995. The basic principle can be expressed as follows: in a D-dimensional search space, a group of random particles is first generated by particle swarm initialization, and the characteristics of the particle are represented by position, velocity, and fitness values. Then, the best solution is found by updating the iteration. In the optimization process, the speed and position of particles are updated by tracking the individual extreme value *p_best_* and the group extreme value *g_best_* [38,39]. The formulas are shown below:

(3) $$V_{id}^{k+1}=\omega V_{id}^{k}+c_{1}r_{1}\left(P_{id}^{k}-X_{id}^{k}\right)+c_{2}r_{2}\left(P_{gd}^{k}-X_{gd}^{k}\right)$$

(4) $$\;\;X_{id}^{k+1}=X_{id}^{k}+V_{id}^{k+1}$$

where ω is the inertia weight; *d* = 1, 2, ..., *D*; *i* = 1, 2, ..., *n*. *k* is the number of current iterations, *V_id_* and *X_id_* are the velocity and position of particles, *c*_1_ and *c*_2_ are nonnegative constants, called acceleration factors. *r*_1_ and *r*_2_ are random numbers distributed between [0, 1].

### 4.5. Model Evaluation

The correct recognition rate (accuracy) of the calibration set and test set for PLS-DA and SVM models was used to evaluate the recognition effect. The higher the value of accuracy, the better the recognition of the model. The formula of accuracy is shown as follows:

(5) $$\mathrm{Accuracy}=\frac{N_{C}}{N_{M}}$$

where *N_C_* represents the number of correctly identified samples, and *N_M_* represents the total number of samples.

## 5. Conclusions

In the study, NIRDRS combined with PLS-DA and SVM methods was adopted to distinguish different types of rice and transgenic/non-transgenic rice. The SG 1st-Der, combined with the SVM, provided the optimal model to distinguish different types of rice. The accuracy of the validation test was 98.33%. For the discrimination of transgenic/non-transgenic rice, the SNV-SVM model was optimal with the accuracy of 100%. Portable NIR spectroscopy, along with chemometrics techniques, could provide another fast and accurate method for identifying rice types and transgenic characteristics (Bt63).

## Figures and Tables

**Table 1:** PLS-DA (partial least squares discriminant analysis) results of the calibration set samples with different pretreatment methods for the identification of rice varieties.

Methods	No. of LVs ^1^	Accuracy (%)
Origin	8	95.83
NWS ^2^	14	97.50
SNV ^2^	7	95.00
MSC ^2^	7	95.00
SG 1st-Der ^2^	11	95.83

^1^ LVs is the abbreviation of latent variable. ^2^ NWS, SNV, MSC, SG 1st-Der are the abbreviations of the spectra pretreatment method Norris–Williams smooth, standard normal variate, multiplicative scatter correction and Savitzky–Golay 1st derivative respectively.

**Table 2:** PLS-DA results of the calibration set samples with different pretreatment methods for the identification of transgenic characteristics.

Methods	No. of LVs	Accuracy (%)
Origin	14	98.75
NWS	20	99.17
SNV	16	100.00
MSC	16	100.00
SG 1st-Der	14	100.00

**Table 3:** SVM (support vector machines) results of the calibration set samples with different pretreatment methods for the identification of rice varieties.

Methods	C ^1^/Gamma	Accuracy (%)
Origin	92.70/3.49	92.70
NWS	34.73/16.78	93.33
SNV	7.81/26.08	98.33
MSC	7.00/969.54	98.75
SG 1st-Der	93.34/1000	99.58

^1^ C represents the tolerance of the SVM model to the error.

**Table 4:** SVM results of the calibration set samples with different pretreatment methods for the identification of transgenic characteristics.

Methods	C/Gamma	Accuracy (%)
Origin	100/10.9	99.38
NWS	100/14.33	99.79
SNV	18.63/35.68	100.00
MSC	17.42/1000	100.00
SG 1st-Der	78.02/1000	99.58

**Table 5:** The number of samples.

Types	Number of Samples
Cambodia Jasmine rice	120
Thai rice	120
Cinnamon soft rice	120
Bt-SY63 rice	360
SY63 rice	360

## Supplementary Materials

The following are available online at https://www.mdpi.com/1420-3049/24/24/4568/s1, The file of supplementary materials contains all the data which the paper used.
